# Long-term drug effectiveness and survival for reference rituximab in rheumatoid arthritis patients in an ordinary outpatient clinic

**DOI:** 10.1038/s41598-022-12271-9

**Published:** 2022-05-18

**Authors:** Katarzyna Łosińska, Mateusz Wilk, Are Hugo Pripp, Mariusz Korkosz, Glenn Haugeberg

**Affiliations:** 1 Division of Rheumatology and Immunology, University Hospital, Krakow, Poland; 2 Oslo Centre of Biostatistics and Epidemiology, Oslo University Hospital, Oslo, Norway; 3 Department of Rheumatology and Immunology, Jagiellonian University Medical College, Krakow, Poland; 4 Division of Rheumatology, Department of Internal Medicine, Sørlandet Hospital, Kristiansand, Norway; 5 Department of Neuromedicine and Movement Science, Faculty of Medicine and Health Sciences, NTNU, Norwegian University of Science and Technology, Trondheim, Norway

**Keywords:** Diseases, Medical research, Rheumatology

## Abstract

To explore the long-term drug effectiveness and survival of reference rituximab (ref-RTX)-treated rheumatoid arthritis (RA) patients in an ordinary outpatient clinic. Second, we explored baseline predictors of drug effectiveness and survival, and third, we clarified reasons for stopping treatment. RA patients treated with ref-RTX between 2006 and 2020 in Norway were examined and monitored using recommended measures for disease activity and patient-reported outcomes (PROs). Drug effectiveness was assessed with random intercept linear mixed models; drug survival was assessed with Kaplan–Meier survival analysis. Reasons for discontinuation were ascertained. Baseline predictors of drug effectiveness and survival were estimated. Among 246 RA patients, at baseline, 17.1% were biologic disease-modifying anti-rheumatic drugs (bDMARDs) naïve, and 51.6% were currently using conventional synthetic DMARDs (csDMARDs). During the five-year follow-up, all disease activity and PRO measures improved significantly (p < 0.01), with more substantial changes noted in the second year. Drug survival was 83% after one year and declined to 34% after five years. The two most frequently reported reasons for discontinuation were the doctor’s decision (36.2%) and lack or loss of effectiveness (19.2%). No significant difference was found between naïve and previous users of bDMARDs or between concomitant and nonconcomitant users of csDMARDs when analysing drug effectiveness and survival. Our real-life data show that ref-RTX-treated RA patients had satisfactory treatment responses; drug survival declined linearly over time. There was no significant difference between naïve and previous users of bDMARDs or between concomitant and nonconcomitant users of csDMARDs, both for drug effectiveness and survival.

## Introduction

In randomized controlled trials (RCTs), reference rituximab (ref-RTX) has been shown to effectively suppress inflammation and reduce structural joint damage in rheumatoid arthritis (RA), especially in patients who are rheumatoid factor (RF)-positive^1^. Ref-RTX is an anti-B cell genetically engineered chimeric monoclonal antibody that binds to the protein CD20 on the surface of B cells, resulting in apoptosis and depletion of these cells. This drug was initially used for the treatment of non-Hodgkin´s lymphoma and in 2006 was approved for the treatment of moderate-to-severe RA in combination with methotrexate (MTX) in patients who had an inadequate response or intolerance to disease-modifying anti-rheumatic drugs (DMARDs), including at least one tumour necrosis factor inhibitor (TNFi)^2^.

In the latest European League Against Rheumatism (EULAR) recommendations for RA management published in 2019, RTX was also recommended as a first-line biological therapy if the treatment target was not achieved with conventional synthetic DMARDs (csDMARDs)^3^.

Data from registries and structured medical records used in outpatient clinics are necessary to establish the long-term efficacy and safety of the ref-RTX drug for the treatment of RA in ordinary clinical practice^4^. For ref-RTX, there is a deficiency of long-term studies exploring drug effectiveness^5–9^ and drug survival^9–13^ in RA patients treated in ordinary clinical practice. As inconsistent findings have been reported in the literature, there is also a need to further illuminate the clinical value of concomitant use of csDMARDs^6,8,12^, the use of ref-RTX as a first-line biologic drug and to explore predictors of the clinical outcome when the drug is used in ordinary clinical practice.

The primary aim of this study was to explore long-term drug effectiveness and survival for ref-RTX in an outpatient cohort of RA patients. The second aim was to explore baseline predictors for drug effectiveness and survival, and the third aim was to illuminate reasons for stopping treatment with ref-RTX.

## Materials and methods

### Data source and study population

The study population included RA patients who were treated with ref-RTX at an ordinary outpatient clinic in Norway between 2006 and 2020. Patients were treated with ref-RTX using standard infusion procedures, including comedication (antihistamine and methylprednisolone). Recommended outcome measures were followed using the clinical GoTreatIt® Rheuma software (https://www.diagraphit.com). The data collection was recorded at clinical visits defined by the treating doctor decision or at time points when the ref-RTX infusion was given. Patients were reported to the computer system as patient-reported outcomes (PROs). Standardised joint counts performed by doctors or trained nurses were also collected along with laboratory and treatment data.

A predefined query was used to retrieve data for ref-RTX from the hospital database. The predefined query displayed data structured on Excel datasheets for baseline, last visit or cessation date and for visits within the one-year period prior to baseline and for subsequent one-year periods after baseline. Patients who commenced treatment less than one year before data extraction were excluded to obtain at least one year of follow-up.

### Data variables

At visits, a broad variety of data was collected. Data variables for this study included age, sex, body mass index (BMI), smoking, disease duration, RF, anti-cyclic citrullinated peptide antibodies (ACPA), C-reactive protein (CRP), erythrocyte sedimentation rates (ESR), 28 swollen and tender joint counts (SJC28 and TJC28), patient global assessment (PGA) reported on 0–100 mm visual analogue scale (VAS), a modified health assessment questionnaire (MHAQ) and composite scores, the disease activity score with 28 joint counts (DAS28 ESR), and the clinical disease activity index (CDAI). The dates of the first and consecutive doses of RTX were also collected, and previously used DMARDs, both conventional synthetic and biological (including their line number), were noted. Concomitant DMARDs, dates of onset and withdrawal, and main reasons for discontinuation were also recorded in the database.

### Study analysis and study endpoints

Analyses for drug effectiveness were based on available data with no imputation of missing data. The percentage of missing data is presented to display data density. Data for effectiveness and survival were analyzed for the whole cohort and groups according to previous bDMARD exposure (bDMARD-naïve, bDMARD-exposed), concomitant csDMARD use (csDMARDs, no csDMARDs), and RF and ACPA status.

Variables for exploring changes over the five-year period in disease activity included CRP, ESR, SJC28, TJC28, DAS28 and CDAI, and for PRO measures PGA and MHAQ. The proportions of patients with good, moderate or no response according to the EULAR response criteria^14^ were also explored. An analysis of the baseline predictors of drug effectiveness and survival was performed.

### Statistics

For the description of the population means and standard deviations (SD), absolute and/or relative frequencies were used. Parametric statistics were used for comparisons of patient characteristics, i.e. the chi-squared test for categorical variables and independent samples t-test for continuous variables. Estimated means during follow-up are presented as the mean and standard error (SE). The difference between the observation times within each treatment group was assessed with random intercept linear mixed models. To lower the risk of results being confounded, confounding variables (concomitant use of csDMARDs, previous use of bDMARDs) were included as covariates in the linear mixed models.

Baseline predictors of drug effectiveness were explored in two complementary approaches. The first was multivariable logistic regression models with estimates calculated at one, two, three, four and five years of follow-up with good or moderate response defined according to the EULAR criteria as a dependent variable. The second was multivariate linear regression models with DAS28 change after 2 years of follow-up as the dependent variable (two-year DAS28 change was selected because of the greatest drop compared to baseline). The investigated baseline variables included demographic factors (sex, age), disease duration, concomitant use of csDMARDs, previous use of bDMARDs, RF and ACPA status, laboratory and clinical measures reflecting disease activity (ESR, TJC28, SJC28 and DAS28) and PGA. To test their consistency and strength, the models were developed via a forward, stepwise and backward variable selection method removing all variables with a p-value greater than 0.10. To avoid collinearity, parallel models were formed, one with the composite DAS28 along with other investigated variables and another with the components of DAS28 (ESR, TJC28, SJC28, PGA) along with other investigated variables. The models were also tested without DAS28. Due to the high correlation coefficient between ESR and CRP, only ESR was included in the analyses. The final model consisted of age, sex, RF and ACPA status, DAS28, concomitant use of csDMARDs, and previous use of bDMARDs.

RTX drug survival rates were described using Kaplan–Meier analysis with estimates calculated at 1, 2, 3, 4 and 5 years of follow-up. Between subgroups, differences were estimated with a log-rank test. Baseline variables associated with drug survival were assessed using multivariable Cox proportional hazard models. The models were developed via a forward, stepwise and backward variable selection method by removing all variables with a p-value greater than 0.10. The investigated baseline variables included demographic factors (sex, age), disease duration, concomitant use of csDMARDs, previous use of bDMARDs, RF and ACPA status, laboratory and clinical measures reflecting disease activity (ESR, TJC28, SJC28 and DAS28) and PGA. To avoid collinearity, parallel models were formed, one with the composite DAS28 along with other investigated variables and another with the components of DAS28 (ESR, TJC28, SJC28, PGA) along with other investigated variables. The models were also tested without DAS28. Due to the high correlation coefficient between ESR and CRP, only ESR was included in the analyses. The final model consisted of age, sex, RF and ACPA status, DAS28, concomitant use of csDMARDs, and previous use of bDMARDs.

All statistical analyses were performed using SAS Studio (SAS Institute, Cary, North Carolina, USA). P-values less than 0.05 were considered significant.

### Ethics

The study was approved by the regional ethical committee (REC; Regional etisk komite Midt-Norge 2010/3078). Regional Committees for Medical and Health Research Ethics—REC Central (REK; Regionale komiteer for medisinsk og helsefaglig forskningsetikk) waived the need for informed consent from patients, as all data were anonymised and collected as part of routine clinical care. The study complied with the Declaration of Helsinki.

## Results

### Baseline characteristics

A total of 249 RA patients were treated with ref-RTX between 31.01.2006 (the first patient started treatment) and 11.05.2020 (date of data extraction). Three patients with a follow-up period shorter than 1 year were excluded.

Group characteristics at baseline are presented in Table 1. For the 246 RA patients eligible for the analysis, 74.8% were females, the mean (SD) age was 59.1 (13.5) years, the disease duration was 13.0 (10.2) years, 88.8% were positive for RF and 92.1% were positive for ACPA. In the majority (82.9%) of patients, first-cycle ref-RTX treatment was given at a dosage of 2000 mg; 127 (51.5%) patients were current users of csDMARDs, and 42 (17.1%) were bDMARD naïve. Mean (SD) values at baseline were CRP 23.1 (33.0) mg/L, ESR 32.1 (22.1) mm/hr, SJC28 6.3 (5.4), TJC28 7.1 (6.8), DAS28 4.9 (1.4), CDAI 22.9 (13.3), PGA on VAS 57.2 (25.5) mm, IGA on VAS 36.9 (22.3) mm, and MHAQ 1.0 (0.6). A statistically significant difference between bDMARD-naïve and bDMARD-exposed patients was only found for disease duration (8.6 vs. 13.9 years, p < 0.01) and between concomitant and nonconcomitant use of csDMARDs for age (65.8 vs. 69.4 years, p = 0.04) and disease duration (10.4 vs. 15.7 years, p < 0.01). More detailed information on baseline group characteristics, including missing data, is shown in Supplementary Table S1.

**Table 1:** Group characteristics at baseline.

	Total cohort (N = 246)	bDMARD-exposed (N = 204)	bDMARD-naïve (N = 42)	p-value (A)	csDMARDs (N = 127)	No csDMARDs (N = 119)	p-value (B)
Age, years	59.1 (13.5)	67.2 (13.6)	61.9 (13.5)	0.1390	65.8 (13.2)	60.9 (13.8)	0.0406
Female, n	184 (74.8%)	156 (76.5%)	28 (66.7%)	0.1827	94 (74.0%)	90 (75.6%)	0.7707
BMI, kg/m^2^	25.8 (5.0)	25.6 (5.0)	26.5 (5.4)	0.4349	26.5 (5.5)	25.0 (4.4)	0.0532
Current smoker, n	49 (19.9%)	42 (20.6%)	7 (16.7%)	0.5623	27 (21.3%)	22 (18.5%)	0.5864
Disease duration, years	13.0 (10.2)	13.9 (10.1)	8.6 (10.0)	0.0024	10.4 (8.3)	15.7 (11.3)	< 0.0001
RF positive, n	214 (88.8%)	177 (88.1%)	37 (92.5%)	0.8154	111 (88.8%)	103 (88.8%)	0.8436
ACPA positive, n	221 (92.1%)	184 (92.0%)	37 (92.5%)	0.9149	117 (93.6%)	104 (90.4%)	0.3643
**First cycle RTX dose, n** 500 mg 1000 mg 1500 mg 2000 mg	1 (0.4%) 38 (15.5%) 3 (1.2%) 204 (82.9%)	1 (0.5%) 32 (15.7%) 1 (0.5%) 170 (83.3%)	0 6 (14.3%) 2 (4.8%) 34 (81.0%)	0.8937^a^	0 19 (15.0%) 0 108 (85.0%)	1 (0.8%) 19 (16.0%) 3 (2.5%) 96 (80.7%)	0.7389^a^
Current csDMARDs ^b^, n	127 (51.6%)	108 (52.9%)	19 (45.2%)	0.3630	127 (100%)	0	< 0.0001
Current MTX, n	97 (39.4%)	85 (41.7%)	12 (28.6%)	0.1138	97 (76.4%)	0	< 0.0001
Current steroids, n	181 (73.6%)	148 (72.6%)	33 (78.6%)	0.4202	87 (68.5%)	94 (79.0%)	0.0623
**Number of prior bDMARDs, n** 0 1 2 3 or more	42 (17.1%) 68 (27.6%) 76 (30.9%) 60 (24.4%)	0 68 (33.3%) 76 (37.3%) 60 (29.4%)	0 0 0 0	< 0.0001	19 (15.0%) 39 (30.7%) 41 (32.2%) 28 (22.1%)	23 (19.3%) 29 (24.4%) 35 (29.4%) 32 (26.9%)	0.5060
CRP, mg/L	23.1 (33.0)	21.6 (26.7)	30.6 (54.4)	0.3128	22.3 (30.7)	23.9 (35.4)	0.7133
ESR, mm/h	32.1 (22.1)	31.8 (21.1)	33.3 (26.1)	0.6916	30.7 (20.8)	33.6 (23.2)	0.3102
DAS28	4.9 (1.4)	4.9 (1.4)	4.9 (1.6)	0.8550	4.8 (1.3)	5.1 (1.6)	0.1178
SJC28, 0–28	6.3 (5.4)	6.4 (5.4)	5.6 (4.9)	0.3763	5.8 (5.3)	6.8 (5.4)	0.1736
TJC28, 0–28	7.1 (6.8)	7.2 (6.9)	6.5 (6.1)	0.5234	6.3 (6.1)	8.0 (7.4)	0.0622
PGA, 0–100 mm	57.2 (25.5)	58.5 (24.5)	51.1 (29.1)	0.0903	56.9 (25.9)	57.6 (25.1)	0.8464
IGA, 0–100 mm	36.9 (22.3)	37.0 (22.4)	6.6 (22.2)	0.9066	5.3 (22.7)	38.7 (21.8)	0.2497
MHAQ, 0–3	1.0 (0.6)	1.0 (0.6)	0.8 (0.6)	0.1181	0.9 (0.6)	1.0 (0.6)	0.5342
CDAI	22.9 (13.3)	23.0 (13.2)	22.4 (13.8)	0.7978	21.6 (12.5)	24.3 (14.0)	0.1319

Continuous data are presented as the means with standard deviations (SD), and categorical variables are presented as numbers and percentages.

(A)   p-value < 0.05 between bDMARD-exposed and bDMARD-naïve subgroups; (B)   p-value < 0.05 between csDMARD and no csDMARD subgroups.

*CRP* C-reactive protein, *ESR* erythrocyte sedimentation rate, *SJC28* 28 swollen joint count, *TJC28* 28 tender joint count, *DAS28* disease activity score with 28 joint counts, *CDAI* clinical disease activity index, *PGA* patient global assessment, *IGA* investigator global assessment, *MHAQ* modified health assessment questionnaire, *bDMARDs* biologic disease-modifying anti-rheumatic drugs, *csDMARDs* conventional synthetic disease-modifying anti-rheumatic drugs, *BMI* body mass index, *RF* rheumatoid factor, *ACPA* anti-cyclic citrullinated peptide antibodies, *MTX* methotrexate.

^a^Calculated for 1000 mg and 2000 mg; ^b^ csDMARDs include MTX, leflunomide, hydroxychloroquine and sulfasalazine.

### Drug effectiveness

Table 2 presents measures of disease activity and PROs during baseline, the one-year period prior to baseline and for five subsequent years after baseline. During follow-up, all disease activity and PRO measures improved significantly in comparison to baseline. The least improvement in these outcomes was seen in the first year of follow-up, with a subsequent largest improvement in the second year, which increased in subsequent years. The treatment effect was maintained during follow-up, i.e., DAS28 was 4.9 at baseline, for the first year 4.7, the second year 3.6, the third year 3.1, the fourth year 2.8, and the fifth year 2.7. Between bDMARD naïve and non-bDMARD naïve patients and between concomitant and non-concomitant users of csDMARDs, no significant differences were seen either prior to baseline, at baseline or in the subsequent years after baseline (data not shown).

**Table 2:** Changes in disease activity and PRO measures for RA patients treated with ref-RTX.

	52 weeks prior to baseline (N = 246)	N	Missing data	0 weeks (baseline) (N = 246)	N	Missing data	0–52 weeks (1 year) (N = 246)	N	Missing data	52–104 weeks (2 years) (N = 204)	N	Missing data	104–156 weeks (3 years) (N = 163)	N	Missing data	156–208 weeks (4 years) (N = 130)	N	Missing data	208–260 weeks (5 years) (N = 111)	N	Missing data	p-value ^a^
CRP, mg/L	15.0 (1.2)	198	19.51%	23.1 (2.1)	239	2.85%	21.3 (1.6)	239	2.85%	11.7 (0.9)	198	2.44%	8.6 (1.4)	151	4.88%	6.2 (0.7)	118	4.88%	6.7 (0.9)	103	3.25%	< 0.01
ESR, mm/hr	26.5 (1.4)	195	20.73%	32.1 (1.4)	239	2.85%	31.1 (1.3)	239	2.85%	22.3 (1.1)	194	4.07%	17.6 (1.2)	145	7.32%	14.3 (1.0)	112	7.32%	13.6 (1.2)	100	4.47%	< 0.01
SJC28, 0–28	4.1 (0.3)	205	16.67%	6.3 (0.4)	238	3.25%	5.4 (0.3)	238	3.25%	3.2 (0.3)	199	2.03%	2.2 (0.2)	154	3.66%	1.6 (0.2)	123	2.85%	1.5 (0.2)	104	2.85%	< 0.01
TJC28, 0–28	4.9 (0.4)	205	16.67%	7.1 (0.4)	238	3.25%	6.6 (0.3)	238	3.25%	3.6 (0.3)	199	2.03%	2.6 (0.3)	154	3.66%	2.2 (0.3)	123	2.85%	1.8 (0.3)	104	2.85%	< 0.01
DAS28	4.2 (0.1)	192	21.95%	4.9 (0.1)	233	5.28%	4.7 (0.1)	233	5.28%	3.6 (0.1)	189	6.10%	3.1 (0.1)	143	8.13%	2.8 (0.1)	110	8.13%	2.7 (0.1)	94	6.91%	< 0.01
CDAI	16.1 (0.8)	199	19.11%	22.9 (0.9)	234	4.88%	20.7 (0.7)	234	4.88%	12.3 (0.7)	193	4.47%	9.4 (0.7)	150	5.28%	8.5 (0.6)	123	2.85%	7.7 (0.7)	101	4.07%	< 0.01
PGA, 0-100 mm	46.7 (1.6)	207	15.85%	57.2 (1.7)	238	3.25%	53.7 (1.4)	238	3.25%	38.1 (1.6)	198	2.44%	33.7 (1.9)	153	4.07%	35.0 (2.1)	126	1.63%	32.8 (2.2)	105	2.44%	< 0.01
IGA, 0-100 mm	24.2 (1.1)	204	17.07%	36.9 (1.5)	238	3.25%	33.2 (1.1)	238	3.25%	19.2 (1.0)	198	2.44%	13.7 (1.1)	152	4.47%	12.1 (0.9)	125	2.03%	11.8 (1.1)	103	3.25%	< 0.01
MHAQ, 0–3	0.7 (0.0)	208	15.45%	1.0 (0.0)	237	3.66%	0.9 (0.0)	237	3.66%	0.7 (0.0)	196	3.25%	0.6 (0.0)	153	4.07%	0.5 (0.0)	121	3.66%	0.5 (0.0)	103	3.25%	< 0.01

Data are presented as the mean with standard error (SE) of the mean.

*PRO* patient-reported outcome, *RA* rheumatoid arthritis, *Ref-RTX* reference rituximab, *CRP* C-reactive protein, *ESR* erythrocyte sedimentation rate, *SJC28* 28 swollen joint count, *TJC28* 28 tender joint count, *DAS28* disease activity score with 28 joint counts, *CDAI* clinical disease activity index, *PGA* patient global assessment, *IGA* investigator global assessment, *MHAQ* modified health assessment questionnaire.

^a^p-value for difference between the observation times (random intercept linear mixed models).

**Figure 1:**
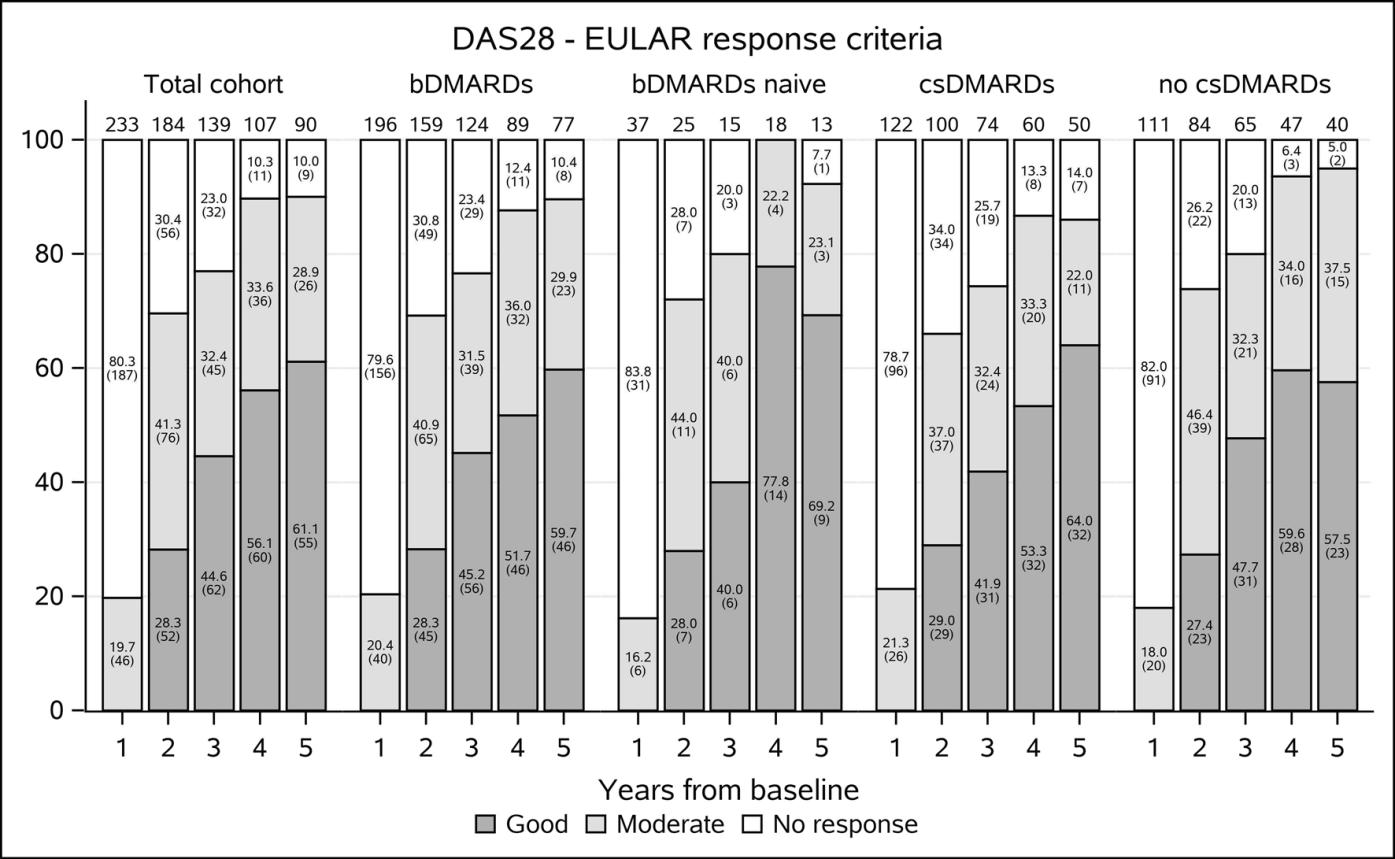
The percentage of rheumatoid arthritis patients treated with reference rituximab with no response, moderate response and good response as defined by EUALR response criteria^13^ throughout the 5-year follow-up period. Data are shown for the whole cohort and for patients stratified according to previous or no previous use of biologic treatment and for concomitant and nonconcomitant use of csDMARDs. No significant difference was found between the subgroups.

The proportions of patients on ref-RTX with no EULAR response were 80.3% in the first year, 30.4% in the second year, 23.0% in the third year, 10.3% in the fourth year and 10.0% in the fifth year. Those with a moderate EULAR response were 19.7%, 41.3%, 32.4%, 33.6%, and 28.9%, and those with a good EULAR response were 0%, 28.3%, 44.6%, 56.1%, and 61.1%. Similar EULAR response rates, a pattern of change in the proportions of good, moderate and nonresponders as well as the largest improvement in the second year of follow-up, were found in an analysis of a subgroup of patients who did not respond after the first year (108 patients with full data available for 3 years of the follow-up). After the second year, more than half (N = 73, 67.6%) of them reached a good or moderate EULAR response, and after the third year, the number of good and moderate EULAR responders was still increasing (N = 81, 75.0%). In Fig. 1, the results for the total cohort and for bDMARD naïve and bDMARD nonnaïve patients and for concomitant and nonconcomitant users of csDMARDs are shown.

### Drug survival

**Figure 2:**
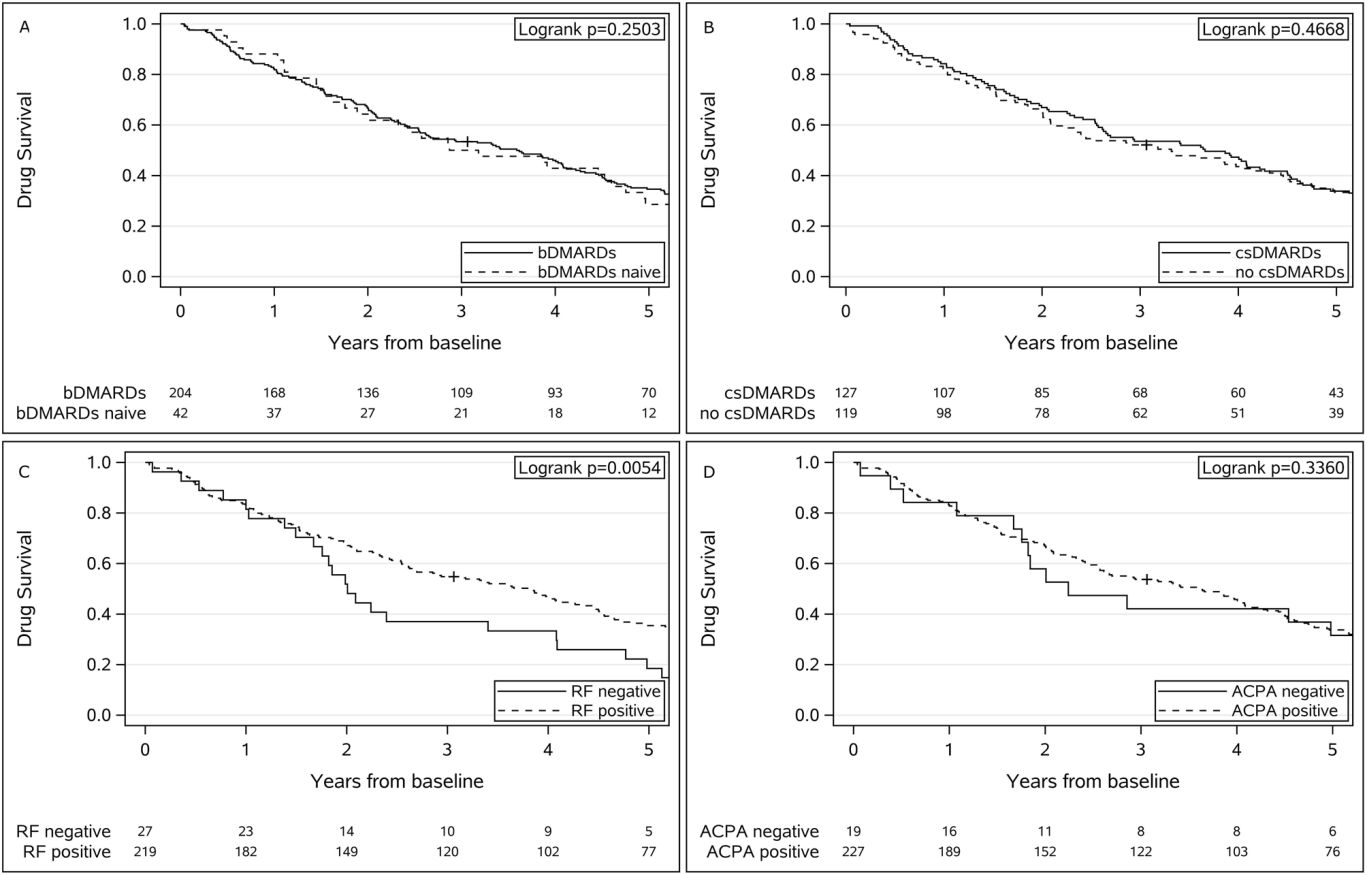
Kaplan–Meier plots of treatment retention rates among rheumatoid arthritis outpatient clinic patients treated with reference rituximab comparing (**A**) naïve and nonnaïve to bDMARDs, (**B**) concomitant and nonconcomitant users of csDMARDs, (**C**) RF positive versus RF negative and (**D**) ACPA positive versus ACPA negative.

Drug survival for ref-RTX was 83% (95% CI 77–87%) after 1 year, 66% (95% CI 60–72%) after 2 years, 53% (95% CI 46–59%) after 3 years, 46% (95% CI 39–52%) after 4 years and 34% (95% CI 28–40%) after 5 years of follow-up. As shown in Fig. 2, no significant differences in drug survival were found between RA patients treated with and without concomitant csDMARDs (p = 0.47), patients naïve to bDMARDs versus previous users of bDMARDs (p = 0.25), or ACPA-positive versus ACPA-negative patients (p = 0.35). However, significantly better drug survival was found in RF-positive versus RF-negative patients (p < 0.01).

### Baseline predictors of treatment outcome

In the logistic regression analysis, the only variable identified to be independently associated with a good or moderate response defined according to the EULAR criteria was high baseline DAS28 for the whole follow-up period (after 1 year, OR 1.741, 95% CL 1.339–2.263; 2 years OR 2.025, 95% CL 1.525–2.689; 3 years, OR 1.890, 95% CL 1.372–2.604; 4 years, OR 2.761, 95% CL 1.533–4.972 and 5 years, OR 3.897, 95% CL 1.700–8.933). In the linear regression analysis, male sex, high baseline DAS28 and ACPA positive status were found to be independently associated with better improvement (defined as higher DAS28 reduction) after the second year of follow-up.

In the prediction analysis, only RF positive status (HR 0.561, 95% CI 0.364–0.864) was found to be independently associated with better drug survival.

### Reasons for drug discontinuation

In the total cohort, the most frequent reason for ref-RTX discontinuation was reported to be doctor’s decision (36.2%), followed by lack or loss of effectiveness (19.2%), adverse effects (14.4%), remission (10.1%), patient’s decision (7.5%), death (3.2%) and others (9.6%). A higher DAS28 score was generally found in patients stopping treatment because of a lack or loss of efficacy of ref-RTX, whereas a lower DAS28 was found where the doctor’s decision or remission were the reasons for cessation. More detailed information on the reasons for stopping ref-RTX treatment and their distribution is displayed in Supplementary Table S2.

## Discussion

The main finding in this study is that in the ref-RTX-treated RA patients, a significant clinical treatment response, both for measures of disease activity and PROs, was achieved primarily in the second year, and the treatment effect was maintained during five years of follow-up. Interestingly, we found no significant differences between bDMARD naïve and previous users of bDMARDs or between concomitant and nonconcomitant users of csDMARDs, both for ref-RTX effectiveness and drug retention rates.

The only variable identified in our study to be independently associated with a good or moderate response according to the EULAR criteria was high baseline DAS28. However, in the linear regression analysis, high baseline DAS28, male sex and ACPA positive status were found to be independently associated with a better improvement in DAS28 after the second year. Our findings are in accordance with other reports where ACPA positive status^15,16^, higher baseline DAS28 and male sex have been reported to predict DAS28 improvement in RTX-treated patients^17^. Despite the fact that the presence of RF has been demonstrated to be associated with a more robust treatment response to RTX in a number of studies^6–8,17,18^, we did not find it to be a predictor of DAS28 reduction.

Studies exploring the link between bDMARD exposure and ref-RTX effectiveness yielded disparate results. Richter et al.^6^ found a higher number of previous TNFi failures to be a significant predictor of poorer response. In contrast, Valleala et al. demonstrated that the number of previously failed TNFis did not predict the response to RTX^7^. Others argued that a better response was noted for TNFi naïve patients^15,17^. These results are in contrast to our study, which showed no difference in treatment response between previous and nonprevious users of bDMARDs. However, the follow-up periods in those studies were 6–12 months, whereas our observation comprised five years of real-life data, which is why the comparison of short-term and long-term data should be performed with caution.

Although the use of ref-RTX in RA patients is approved only in combination with MTX, ref-RTX monotherapy or administration with concomitant csDMARDs other than MTX is frequent in daily clinical practice. Canamares et al.^18^ reported an increasing number of ref-RTX monotherapy RA patients (from 11% to nearly 24%) in recent years. Our study demonstrating similar ref-RTX effectiveness while used with or without csDMARDs is consistent with previously published real-life data^5,6^ and should be of value to clinicians when selecting the optimal treatment strategy for RA patients commencing RTX. There is also a growing number of patients treated with ref-RTX as their first bDMARD, usually due to contraindications to TNFi or cost-saving potential^19^. The percentage of patients treated with ref-RTX as a first-line bDMARD in our cohort was approximately 20%, which is in line with other reports ranging from 16 to 28%^5,7,8,11,13,17,19^.

Considering PROs, significant gradual reductions in PGA and MHAQ were seen over the observed period. Improvement in HAQ scores was previously noticed by others^5,7,8^, but a reduction in PGA was to date reported in only one study^7^.

The optimal treatment regimen for RTX in RA has not been definitively determined. The.

most commonly applied course of RTX consists of two 1000 mg intravenous infusions with a two-week interval between each dose, followed by retreatment after 24 weeks or on demand during disease flares. In our study, no good responders according to the EULAR response criteria were noted after the first year of treatment, suggesting that RTX is a long-acting drug where at least two twin infusions should be given before declaring treatment failure.

Drug survival rates are a good proxy measurement of treatment effectiveness, safety and tolerability. Our real-life data show that drug survival for ref-RTX-treated RA patients declines rather linearly over time, and after four years, 46% of the patients were still on the drug. The continuation rates in our study are slightly lower than those previously reported, ranging from approximately 50%^10^ to 59% after four years^11^ and 46.0% after five years^13^. In our prediction analysis, only RF-positive status was found to be independently associated with better drug survival, which has also been reported by others^10–13^.

In contrast to other studies, we did not find a difference in drug survival for patients with or without concomitant use of csDMARDs and previous use of bDMARDs. Canamares et al.^12^ reported that the use of ref-RTX in combination with csDMARDs was associated with better drug persistence. In a study by Oldroyd et al.^11^, the ref-RTX continuation estimate after four years was slightly higher for the bDMARD naïve cohort (65%) vs. the bDMARD exposed cohort (59%). In a retrospective study by Norris-Grey et al.^13^, the ref-RTX treatment continuation rate was lower in those patients who had previously failed at least one bDMARD; in the Cox regression analysis, they found a higher number of previous bDMARDs to be associated with an increased risk of discontinuation. We do not have a convincing explanation for this interesting discrepancy found in our study. However, we might suppose that it rests within the baseline characteristics of patients where a difference between bDMARD naïve and bDMARD exposed was only found for disease duration and between concomitant and nonconcomitant use of csDMARDs for age and disease duration. There were no baseline differences between these groups in disease activity, which could additionally be at play when examining drug survival. A higher DAS28 score was found in patients discontinuing treatment due to a lack or loss of efficacy, and a lower DAS28 was found where the doctor’s decision or remission were the reasons for cessation. Thus, we assume that disease activity, comparable between groups at baseline, was not attenuated by ref-RTX in a proportion of patients during the study regardless of their bDMARD and csDMARD baseline status.

To date, the most frequently reported reason for ref-RTX discontinuation is its inefficacy^7,9–11,19^, ranging from 18%^19^ to 73%^9^. However, in our study, the most frequent reason for ref-RTX treatment cessation was the doctor’s decision (36.2%), followed by lack or loss of effectiveness (19.2%). These two were different with regard to DAS28, as mentioned above.

Our study should be seen in the context of its limitations. For all observational studies, there are issues related to a certain level of missing data, confounding factors and attrition bias. The single-center character and a lack of comparison of RTX effectiveness and survival with other bDMARDs, i.e., TNFi, are also important weaknesses of this study. The absence of validated measures defining treatment failure as well as the fact that as many as one-third of the reasons for treatment termination were assigned to the unspecified “doctors’ decision” should also be considered relevant limitations. These drawbacks are somewhat balanced by the longest reported follow-up to date a substantial number of patients treated in a real-life setting. Patients were monitored with recommended outcome measures during appointment intervals according to local standards of care, thus reflecting real-life use of ref-RTX in the RA cohort and providing proof in practice data. The strength of the study is the real-life setting, reporting of data prior to treatment start and the robust data density with a low percentage of patients with missing data at the visits.

The clinical implications of the study are that our findings may support ref-RTX use as monotherapy without csDMARD comedication and that a similar treatment response could be achieved independently of prior bDMARD therapy. We also recommend at least two twin infusions of ref-RTX with a 6-month interval, followed by an observation period for at least one year, before identifying treatment failure.

To conclude, our real-life 5-year data revealed that ref-RTX-treated RA patients had a substantial clinical treatment response and drug survival. In overall, results of our study are in line with previous reports and confirm findings from earlier studies. We found no significant differences between bDMARD naïve and previous users of bDMARDs or between concomitant and nonconcomitant users of csDMARDs, both for ref-RTX effectiveness and drug retention rates. RF seropositivity, unlike ACPA, was not predictive of drug effectiveness, but in line with others, RF presence was independently associated with better drug survival. A significant treatment response was seen primarily in the second year, indicating that at least two twin infusions should be given before declaring treatment failure.

## Supplementary Information

Supplementary Tables.

## Data Availability

The data underlying this article will be shared upon reasonable request to the corresponding author.
