# Supplementary material for: Long-term drug effectiveness and survival for reference rituximab in rheumatoid arthritis patients in an ordinary outpatient clinic
Source: Sci Rep. 2022 May 18;12:8283. doi: 10.1038/s41598-022-12271-9 (PMC9117312; doi:10.1038/s41598-022-12271-9)
Supplement: Supplementary file 1 — Supplementary Tables. [file 41598_2022_12271_MOESM1_ESM.docx]

**Long-term drug effectiveness and survival for reference rituximab in rheumatoid arthritis patients in an ordinary outpatient clinic**

Katarzyna Łosińska1*, Mateusz Wilk1, Are Hugo Pripp2, Mariusz Korkosz1, 3, Glenn Haugeberg4, 5

1University Hospital, Division of Rheumatology and Immunology, Krakow, Poland

2Oslo University Hospital, Oslo Centre of Biostatistics and Epidemiology, Oslo, Norway

3Jagiellonian University Medical College, Department of Rheumatology and Immunology, Krakow, Poland

4Sørlandet Hospital, Division of Rheumatology, Department of Internal Medicine, Kristiansand, Norway

5NTNU, Norwegian University of Science and Technology, Department of Neuromedicine and Movement Science, Faculty of Medicine and Health Sciences, Trondheim, Norway

*Correspondence to:

Katarzyna Łosińska

Division of Rheumatology and Immunology

University Hospital

Jakubowskiego 2

30-688 Krakow

Poland

Phone: +48124003100

E-mail: [klosinska@su.krakow.pl](mailto:klosinska@su.krakow.pl)

**Supplementary material**

Supplementary Table S1. Group characteristics at baseline.

|  | **Total cohort**  **(N=246)** | | **bDMARD-exposed**  **(N=204)** | | **bDMARD-naïve**  **(N=42)** | | **p-value**  **(A)** | **csDMARDs**  **(N=127)** | | **no csDMARDs**  **(N=119)** | | **p-value**  **(B)** |
| --- | --- | --- | --- | --- | --- | --- | --- | --- | --- | --- | --- | --- |
|  |  | **Missing data** |  | **Missing data** |  | **Missing data** |  |  | **Missing data** |  | **Missing data** |  |
| Age, years | 59.1 (13.5) | 0% | 67.2 (13.6) | 0% | 61.9 (13.5) | 0% | 0.1390 | 65.8 (13.2) | 0% | 60.9 (13.8) | 0% | 0.0406 |
| Female, n | 184 (74.8%) | 0% | 156 (76.5%) | 0% | 28 (66.7%) | 0% | 0.1827 | 94 (74.0%) | 0% | 90 (75.6%) | 0% | 0.7707 |
| BMI, kg/m^2^ | 25.8 (5.0) | 36.99% | 25.6 (5.0) | 38.73% | 26.5 (5.4) | 28.57% | 0.4349 | 26.5 (5.5) | 33.07% | 25.0 (4.4) | 41.18% | 0.0532 |
| Current smoker, n | 49 (19.9%) | 0% | 42 (20.6%) | 0% | 7 (16.7%) | 0% | 0.5623 | 27 (21.3%) | 0% | 22 (18.5%) | 0% | 0.5864 |
| Disease duration, years | 13.0 (10.2) | 0% | 13.9 (10.1) | 0% | 8.6 (10.0) | 0% | 0.0024 | 10.4 (8.3) | 0% | 15.7 (11.3) | 0% | <0.0001 |
| RF positive, n | 214 (88.8%) | 2.03% | 177 (88.1%) | 1.47% | 37 (92.5%) | 4.76% | 0.8154 | 111 (88.8%) | 1.57% | 103 (88.8%) | 2.52% | 0.8436 |
| ACPA positive, n | 221 (92.1%) | 2.44% | 184 (92.0%) | 1.96% | 37 (92.5%) | 4.76% | 0.9149 | 117 (93.6%) | 1.57% | 104 (90.4%) | 3.36% | 0.3643 |
| First cycle RTX dose, n  500 mg  1000 mg  1500 mg  2000 mg | 1 (0.4%)  38 (15.5%)  3 (1.2%)  204 (82.9%) | 0% | 1 (0.5%)  32 (15.7%)  1 (0.5%)  170 (83.3%) | 0% | 0  6 (14.3%)  2 (4.8%)  34 (81.0%) | 0% | 0.8937^a^ | 0  19 (15.0%)  0  108 (85.0%) | 0% | 1 (0.8%)  19 (16.0%)  3 (2.5%)  96 (80.7%) | 0% | 0.7389^a^ |
| Current csDMARDs ^b^, n | 127 (51.6%) | 0% | 108 (52.9%) | 0% | 19 (45.2%) | 0% | 0.3630 | 127 (100%) | 0% | 0 | 0% | <0.0001 |
| Current MTX, n | 97 (39.4%) | 0% | 85 (41.7%) | 0% | 12 (28.6%) | 0% | 0.1138 | 97 (76.4%) | 0% | 0 | 0% | <0.0001 |
| Current steroids, n | 181 (73.6%) | 0% | 148 (72.6%) | 0% | 33 (78.6%) | 0% | 0.4202 | 87 (68.5%) | 0% | 94 (79.0%) | 0% | 0.0623 |
| Number of prior bDMARDs, n  0  1  2  3 or more | 42 (17.1%)  68 (27.6%)  76 (30.9%)  60 (24.4%) | 0% | 0  68 (33.3%)  76 (37.3%)  60 (29.4%) | 0% | 0  0  0  0 | 0% | <0.0001 | 19 (15.0%)  39 (30.7%)  41 (32.2%)  28 (22.1%) | 0% | 23 (19.3%)  29 (24.4%)  35 (29.4%)  32 (26.9%) | 0% | 0.5060 |
| CRP, mg/L | 23.1 (33.0) | 2.85% | 21.6 (26.7) | 2.45% | 30.6 (54.4) | 4.76% | 0.3128 | 22.3 (30.7) | 2.36% | 23.9 (35.4) | 3.36% | 0.7133 |
| ESR, mm/h | 32.1 (22.1) | 2.85% | 31.8 (21.1) | 2.45% | 33.3 (26.1) | 4.76% | 0.6916 | 30.7 (20.8) | 2.36% | 33.6 (23.2) | 3.36% | 0.3102 |
| DAS28 | 4.9 (1.4) | 5.28% | 4.9 (1.4) | 3.92% | 4.9 (1.6) | 11.90% | 0.8550 | 4.8 (1.3) | 3.94% | 5.1 (1.6) | 6.72% | 0.1178 |
| SJC28, 0-28 | 6.3 (5.4) | 3.25% | 6.4 (5.4) | 2.45% | 5.6 (4.9) | 7.14% | 0.3763 | 5.8 (5.3) | 2.36% | 6.8 (5.4) | 4.20% | 0.1736 |
| TJC28, 0-28 | 7.1 (6.8) | 3.25% | 7.2 (6.9) | 2.45% | 6.5 (6.1) | 7.14% | 0.5234 | 6.3 (6.1) | 2.36% | 8.0 (7.4) | 4.20% | 0.0622 |
| PGA, 0-100mm | 57.2 (25.5) | 3.25% | 58.5 (24.5) | 3.43% | 51.1 (29.1) | 2.38% | 0.0903 | 56.9 (25.9) | 2.36% | 57.6 (25.1) | 4.20% | 0.8464 |
| IGA, 0-100mm | 36.9 (22.3) | 3.25% | 37.0 (22.4) | 2.94% | 36.6 (22.2) | 4.76% | 0.9066 | 35.3 (22.7) | 2.36% | 38.7 (21.8) | 4.20% | 0.2497 |
| MHAQ, 0-3 | 1.0 (0.6) | 3.66% | 1.0 (0.6) | 2.94% | 0.8 (0.6) | 7.14% | 0.1181 | 0.9 (0.6) | 2.36% | 1.0 (0.6) | 5.04% | 0.5342 |
| CDAI | 22.9 (13.3) | 4.88% | 23.0 (13.2) | 4.41% | 22.4 (13.8) | 7.14% | 0.7978 | 21.6 (12.5) | 3.15% | 24.3 (14.0) | 6.72% | 0.1319 |

Continuous data are presented as the means with standard deviations (SD), and categorical variables are presented as numbers and percentages.

A = p-value <0.05 between bDMARD-exposed and bDMARD-naïve subgroups; B = p-value <0.05 between csDMARD and no csDMARD subgroups.

^a^ Calculated for 1000 mg and 2000 mg; ^b^ csDMARDs include MTX, leflunomide, hydroxychloroquine and sulfasalazine.

CRP: C-reactive protein. ESR: erythrocyte sedimentation rate. SJC28: 28 swollen joint count. TJC28: 28 tender joint count. DAS28: disease activity score with 28 joint counts. CDAI: clinical disease activity index. PGA: patient global assessment. IGA: investigator global assessment. MHAQ: modified health assessment questionnaire. bDMARDs: biologic disease-modifying anti-rheumatic drugs. csDMARDs: conventional synthetic disease-modifying anti-rheumatic drugs. BMI: body mass index. RF: rheumatoid factor. ACPA: anti-cyclic citrullinated peptide antibodies. MTX: methotrexate

Supplementary Table S2. Reasons for ref-RTX treatment discontinuation and DAS28 at cessation in RA patients.

| Missing data (%) | **Total cohort**  **(N=246)**  23.6 | **DAS28** | **bDMARD-exposed**  **(N=204)**  20.6 | **DAS28** | **bDMARD-naïve**  **(N=42)**  38.1 | **DAS28** | **csDMARDs**  **(N=127)**  25.2 | **DAS28** | **no csDMARDs**  **(N=119)**  21.9 | **DAS28** |
| --- | --- | --- | --- | --- | --- | --- | --- | --- | --- | --- |
| MD decision, n (%) | 68 (36.2) | 2.7 (1.3) | 58 (35.8) | 2.7 (1.3) | 10 (38.5) | 2.6 (1.2) | 30 (31.6) | 2.8 (1.4) | 38 (40.9) | 2.7 (1.2) |
| Lack or loss of efficacy, n (%) | 36 (19.2) | 4.6 (1.4) | 34 (21.0) | 4.6 (1.5) | 2 (7.7) | 4.3 (0.5) | 23 (24.2) | 4.6 (1.4) | 13 (14.0) | 4.6 (1.5) |
| Adverse effect, n (%) | 27 (14.4) | 3.6 (1.5) | 25 (15.4) | 3.7 (1.5) | 2 (7.7) | 3.0 (1.0) | 13 (13.7) | 3.5 (1.6) | 14 (15.1) | 3.7 (1.5) |
| Remission/no need, n (%) | 19 (10.1) | 2.5 (1.4) | 14 (8.6) | 2.4 (1.2) | 5 (19.2) | 2.8 (1.9) | 11 (11.6) | 2.7 (1.1) | 8 (8.6) | 2.3 (1.7) |
| Patient decision, n (%) | 14 (7.5) | 4.1 (1.2) | 11 (6.8) | 4.1 (1.1) | 3 (11.5) | 4.2 (1.5) | 6 (6.3) | 4.2 (1.2) | 8 (8.6) | 4.1 (1.2) |
| Death, n (%) | 6 (3.2) | 2.3 (0.4) | 4 (2.5) | 2.3 (0.6) | 2 (7.7) | 2.3 (0.1) | 4 (4.2) | 2.2 (0.6) | 2 (2.2) | 2.5 (0.1) |
| Other, n (%) | 18 (9.6) | 2.7 (1.2) | 16 (9.9) | 2.7 (1.3) | 2 (7.7) | 2.5 (0.3) | 8 (8.4) | 2.8 (0.9) | 10 (10.8) | 2.7 (1.4) |

Mean (SD) DAS28 values for all subgroups are displayed.

Ref-RTX: reference rituximab. DAS28: disease activity score with 28 joint counts. RA: rheumatoid arthritis. bDMARD: biologic disease-modifying anti-rheumatic drug. csDMARDs: conventional synthetic disease-modifying anti-rheumatic drugs
